# Design of a Module for Cultivation of Tuberous Plants in Microgravity: The ESA Project “Precursor of Food Production Unit” (PFPU)

**DOI:** 10.3389/fpls.2020.00417

**Published:** 2020-05-15

**Authors:** Roberta Paradiso, Antonio Ceriello, Antonio Pannico, Salvatore Sorrentino, Mario Palladino, Maria Giordano, Raimondo Fortezza, Stefania De Pascale

**Affiliations:** ^1^Department of Agricultural Sciences, University of Naples Federico II, Naples, Italy; ^2^Satellite Systems and Operations - Navigation and Science, Organisation Unit, Telespazio S.p.A., Naples, Italy

**Keywords:** potato, *Solanum tuberosum* L., Bioregenerative Life-Support Systems (BLSSs), hydroponics, cultivation substrate, hydrological characterization, porous tube

## Abstract

Plant cultivation systems for Bioregenerative Life-Support Systems in Space developed on Earth need to be tested in space, where reduced gravity alters the liquid and gas behavior both within the plant and between the plant and its surrounding environment, making the distribution of water and nutrients a critical issue. The ESA project “Precursor of Food Production Unit” (PFPU) aims to design a modular cultivation system for edible tuberous plants (such as potato and sweet potato) in microgravity, to be preliminary tested in ground conditions in the view of successive space application. Among the different modules of the PFPU demonstrator, the Root Module (RM) is the component physically hosting the plant and accommodating tubers and roots. This paper describes the step-by-step procedure adopted to realize the RM, including the design, the building, and the ground testing of its prototype. Specifically, the hydrological characterization of possible cultivation substrates, the set-up of the water distribution system, and the validation test of the assembled prototype in a tuber-to-tuber growing cycle of potato plants are described. Among six substrates tested, including three organic materials and three synthetic materials, cellulosic sponge was selected as the best one, based on the hydrological behavior in terms of air and water transport and water retention capacity. The water sensor WaterScout was successfully calibrated to monitor the water status in cellulosic sponge and to drive irrigation and fertigation management. The designed porous tubes-based distribution system, integrated with water sensors, was able to provide water or nutrient solution in a timely and uniform way in cellulosic sponge.

## Introduction

Higher plants play a key role in Bioregenerative Life-Support Systems (BLSSs) for long-term manned missions in space, by regenerating air through photosynthetic CO_2_ absorption and O_2_ emission, recovering water through transpiration, and recycling human waste products through mineral nutrition (Hendrickx and Mergeay, 2007; Wheeler, 2010). In addition, plants could provide fresh food to integrate into the crew’s diet and help to preserve the astronauts’ wellbeing (Lasseur et al., 2010).

Although plant cultivation for food production is mainly envisaged for long-term missions beyond Low Earth Orbit (LEO), current missions in LEO provide a relevant testbed for technological demonstrators of food complement production. Indeed, any cultivation system evaluated on Earth needs to be tested in space, where weightlessness or reduced gravity alter the liquid and gas behavior, making the distribution of water and the control of moisture in the plant root zone a critical issue, as well as the water flow within the plant and between the plant and its surrounding environment (Porterfield, 2002).

Plant growth, yield, and quality are very dependent on physical and chemical characteristics of the root environment, as a consequence providing favorable conditions to the root system is essential to support a successful cultivation. However, unique growing procedures are needed to effectively cultivate plants in space. Indeed, a nutrient delivery system must provide adequate amounts and uniform distribution of water, nutrients, and oxygen in the root zone, in the presence of a small rooting volume and in microgravity conditions, that make the water behavior unpredictable in both the growing medium and the plant tissue (Porterfield, 2002). In addition, since substrate hydraulic properties in space are not easily deducible from our knowledge of Earth, the development of a reliable plant growth system should be based on the understanding of fluid distribution in porous media under microgravity (Reddi et al., 2005). In this scenario, the interfacial or capillary phenomena, occurring between the nutrient solution and the substrate, become increasingly important in determining the movement of liquid, and may become dominant in microgravity (Ostrach, 1982). For this reason, the water retention curve and transport properties of the substrates are fundamental to predict the water flow and the water and O_2_ availability. Saturated hydraulic conductivity (K_s_) and water retention curves represent the crucial hydrologic characteristics to be considered for adequate substrate selection.

In container-grown plants, the features of the pot-substrate system (i.e., size and shape of the pot and type, volume, and structure of the substrate) strongly influence the nutrient solution holding capacity, determining the mineral and water availability for plant uptake and affecting the rate of root development and the root system architecture (Jones et al., 2012; Heinse et al., 2015). Particularly, small pots and cohesive substrates can ultimately result in root restriction, impeding the proper plant growth, with stronger effects on tuberous and taproot plants. Various gelling agents containing predetermined amounts of water and nutrients have been successfully used in passive plant nutrient delivery systems for brief stays in orbit (Goins et al., 1997). However, for extended cultivation periods, growing media will require more than an initial loading of these inputs, due to the greater plant water consumption and nutrient uptake. Past research in microgravity has focused on a few limited applications of liquid transfer at low matric potentials within coarse-grained porous materials, such as zeolite, arcillite, floral foam, or perlite (Ivanova and Dandolov, 1992; Morrow et al., 1994; Podolsky and Mashinsky, 1994; Levine et al., 1998; Hoehn et al., 2000, 2003).

The tuberous species potato (*Solanum tuberosum* L.) and sweet potato (*Ipomoea batatas* L.) are candidate crops for space cultivation in BLSSs, based on technical and dietary criteria, including environmental requirements, yield potential, and nutritional value (Hoff et al., 1982). Specifically, potato is a highly productive crop, having an elevated harvest index (0.7–0.8, as ratio of edible part to total biomass per plant), and with tubers that constitute an excellent source of carbohydrate and a good source of protein. Cultivation of potato presents several advantages over other crops, such as the availability of numerous cultivars (cultivated varieties) with a wide range of characteristics, the low requirement of fertilizer, the suitability of tubers to be processed quickly with relatively low energy demand, the easily degradable inedible waste, and the possibility to harvest tubers gradually, providing continuous food supply. Within the tuberous species, potato produces stem tubers, easier to harvest compared to the root tubers of sweet potato, and forms more compact aerial parts.

The response of potato plants to the growth in different hydroponic systems (e.g., nutrient film technique, aeroponics) and conditions of cultivation (e.g., nutrient solution composition) and environment (e.g., light intensity, photoperiod, air CO_2_ concentration) have been investigated in Space-oriented ground studies in growth chambers from both the National Aeronautics and Space Administration (NASA; reviewed by Wheeler, 2006, 2009) and the European Space Agency (ESA; Molders et al., 2012; Paradiso et al., 2018), in cultivars selected for cultivation in BLSSs.

Most of the studies aiming to characterize crop production under controlled conditions, in the context of BLSSs, have been conducted using hydroponic culture, with recirculating nutrient solution (closed system) (Wheeler, 2006; Monteiro Corrêa et al., 2008; Molders et al., 2012). However, the root growth of tuberous plants in hydroponics can be limited because of water logging and poor root aeration. Nevertheless, successful ground applications demonstrated the possibility to grow tuberous plants hydroponically, for example by exploiting the nutrient film technique (NFT), with water flowing in the bottom portion of the growth tray (e.g., on opportune spreading mats), avoiding the submersion of the tubers to prevent the development of fungal agents of rottenness (Molders et al., 2012). This arrangement implies the possibility to separate a wetted root zone and an aerated tuber zone. However, for the target microgravity application, separating root and tuber zones is difficult to realize, due to difficulties of water containment and lack of density-driven separation of the liquid and gas phases. As a consequence, cultivation of tuberous plants in microgravity will require alternative systems based on the selection of suitable inert substrates, the development of a no-mixed phase system, and the design of specific nutrient delivery systems to ensure adequate plant growth and high tuber yield.

On these bases, within the activities of “Phase 3 Ground Demonstration” of the ESA Programme MELiSSA (Micro Ecological Life-Support System Alternative), the project “Precursor of Food Production Unit (PFPU) – Phase A System Study” aims to design a modular food complement production unit for the cultivation of edible tuberous plants (i.e., potato and sweet potato) in microgravity. Specifically, the objective is to realize a demonstrator to be preliminary tested in ground conditions, in the view of successive spaceflight experiments in microgravity, on board the International Space Station (ISS) (Thales Alenia Space, 2014).

Within the entire PFPU demonstrator, three key modules are designed: the Root Module, the Nutrient Module, and the Microbial Contamination Control Module (Thales Alenia Space, 2014). Among these, the Root Module is the module physically maintaining the plant, accommodating roots and tubers, assuring the separation between the root zone and the aerial zone, and providing the capability to measure the environmental conditions in the rhyzosphere (e.g., temperature, moisture).

The aim of this paper is to describe the subsequent steps of design, realization, and ground testing of the Root Module prototype. Specifically, the hydrological characterization of possible cultivation substrates, the set-up of the water distribution system, and the validation test of the assembled prototype in a tuber-to-tuber growing cycle of potato plants are reported.

## Materials, Equipment, and Methods

### Preliminary Definition of Root Module Requirements

The design of a payload for experiments on board of the ISS has to consider all the constraints coming from the specific environment. For instance, resources aboard the ISS are limited and have to be shared among both several experiments running in parallel and the ISS’ equipment itself. Crew time is also a limited resource. Therefore, the design of the Root Module must minimize the need for crew operations, and must be easy, quick, and safe to maintain. Accordingly, a plug-and-play approach, with a system that is pre-assembled on ground and requires, once on orbit, a fast configuration for operations (functioning, inspection, and disposal) has to be used.

**FIGURE 1:**
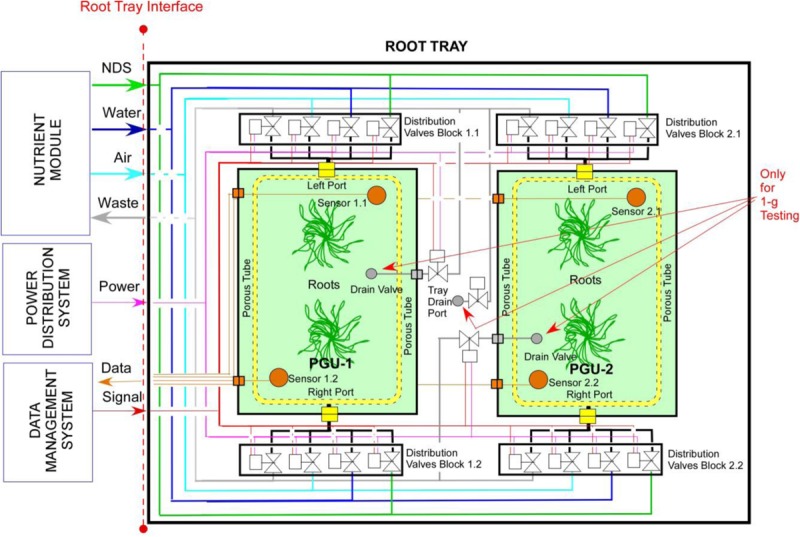
Layout of the root module (RM) and plant growth unit (PGU) root module (RM) designed in the ESA project “precursor of food production unit” (PFPU).

**FIGURE 2:**
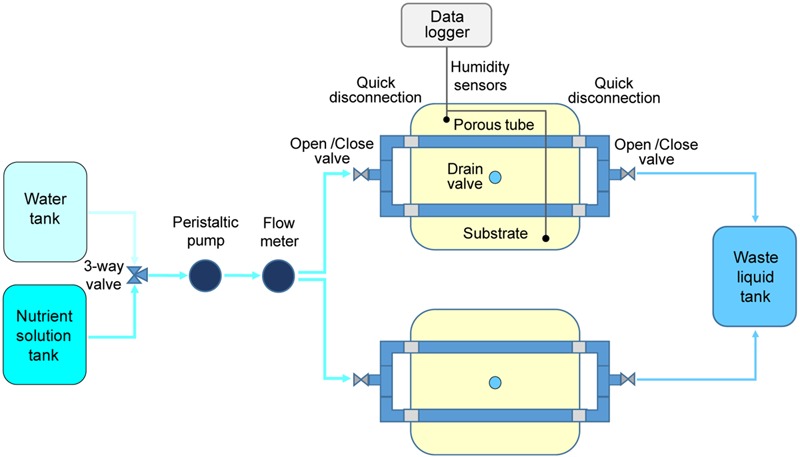
Schematic representation of the system layout adopted in the plant growth test on potato cultivar “Colomba” for validation of (PFPU) Root Module concept.

Following the requirements analysis, the Root Module (RM) was conceived in two parts, namely the Root Tray (RT) and one or more Plant Growth Units (PGUs) (Figure 1). RT is a drawer accommodating the services needed by the PGUs and interfacing other subsystems; the PGU is a bag or a box devoted to host the substrate and the plants (Figure 1). In particular, the RT represents the reusable part of the system and is conceived to support more than one single experiment or mission. It is equipped with all the utilities necessary to forward the resources (power, data, water) from the Power Distribution System and Data Management System to the PGUs. The PGU is conceived as a zip lock Kevlar bag, with a zip on the top part for tubers and roots inspection, and is equipped with porous tubes, embedded in the substrate and connected to an active Nutrient Delivery System (NDS), to distribute the water or nutrient solution to the root zone. In addition, the PGU is equipped with sensors and actuators (electro valves), allowing an automatic management of water and nutrients supply: as soon as the sensors readings (measuring the water content in the substrate) fall below a threshold value, the electro valves are activated to activate an irrigation/fertigation pulse and restore the desired moisture value (Figure 2).

Sensor calibration is a crucial aspect for system set-up, as most of the water sensors are calibrated in natural soils or peat-based substrates. For this reason, a calibration procedure was performed preliminarily to verify the possibility of using commercial sensors for the selected substrate and to build calibration curves to drive irrigation/fertigation in the subsequent cultivation test.

### Test Definition

Following the concept definition, the breadboarding phase aimed to demonstrate the technical feasibility of the identified configuration and at the validation of the concept. The results included the identification of the best cultivar and the best substrate, as well as of the critical aspects in the view of successive tests onboard of the Space Stations.

Based on a step-by-step approach, a sequence of tests was identified as follow:

Phase 1 (Laboratory): Substrates hydrological characterization, aimed to investigate the hydrological behavior of the proposed materials;
Phase 2 (Laboratory): Sensors calibration and Water distribution system set-up, aimed respectively to calibrate moisture sensors and to test the designed porous tubes system for distribution of water and nutrient solution in the selected substrate;
Phase 3 (Growth chamber): Tuber seeds germination and plant growth, aimed to identify the best substrate for potato plants during the early phases of development (tuber sprouting and seedling establishment), and to verify the plant ability to complete a tuber-to-tuber cycle in the proposed system layout.

### Phase 1: Substrates Hydrological Characterization

Six substrates were investigated, including 3 synthetic materials – Oasis Horticubes^®^ (phenolic foam), rockwool and capillary mat (polyester fiber) – and 3 organic materials – cellulosic sponge (100% cellulose), cotton wool and a combination of hemp (*Cannabis sativa* L.) and kenaf (*Hibiscus cannabinus* L.) fibers.

A set of measurements was implemented to determine the hydrological properties, including the saturated hydraulic conductivity and the water retention curves, in these substrates.

To measure saturated hydraulic conductivity (K_s_), substrate samples were packed in cylindrical samplers of 0.05 m length and 0.05 m diameter (3 replicates for each substrate). According to the structural characteristics of each substrate, and in particular based on the presence or absence of a fixed geometry, the sample assemblage was made preserving the native bulk density (BD), or assuring a value permitting gas and water movements. K_s_ was determined using a constant or a falling head permeameter, depending on the velocity of the water flux inside the sampler during the measurement (Reynolds et al., 2002). The characteristic of the porous medium of being passed through by air is defined as air permeability; the latter is quantitatively described by the coefficient k_a_, governing the process of convective transport and transmission of air based on Darcy law (Grant and Groenevelt, 2007). The importance of K_s_ determination also holds a strong positive correlation with permeability k_a_ at a matric potential of −0.05 and −0.10 bar, as evidenced by Loll et al. (1999) and Iversen et al. (2001).

The water retention curve describes the water retention characteristic of the substrate sample subjected to increasing tension and represents the relation between the water content and the water potential. Pressure plate extractor apparatus was adopted on 6 replicates for each substrate and at prefixed values of pressure head (0.2, 0.5, 0.75, 1.0, 1.5, and 2.0 bar) (Dane and Hopmans, 2002). From the analysis of the water retention curves, substrate total porosity can be estimated, assuming for it the value of saturated water content (Kirkham, 2005). It is also possible to calculate water content of the substrate at field capacity, which represents the volume of water retained after the end of drainage and describes the upper limit of water available for the plant, calculating it from the water content at the matric potential of −0.1 bar (Townend et al., 2001). In this work we measured the effective container capacity corresponding to the water held by the substrate against the gravity, considering the effects of the shape and size of the container used, following the methodology of Klute (1986). This measure suggests the proper irrigation volume, reducing the risk of over-irrigation and water excess in the root zone (Sammons and Struve, 2008), taking into account that in long term cultivation experiments in root modules designed for microgravity conditions, a minimum of 10% air-filled porosity is necessary for adequate aeration (Jones and Or, 1998).

### Phase 2: Sensors Calibration and Water Distribution System Set-Up

Based on the substrate hydrological characterization performed in Phase 1, cellulosic sponge was selected as the most suitable substrate for plant growth. Accordingly, Phase 2 aimed to evaluate the substrate/porous tubes integrated system, by verifying the capability of porous tubes to correctly distribute water in cellulosic sponge. The test consisted of two steps: (A) sensor calibration, aimed to build the calibration curve of a selected water sensor in cellulosic sponge; (B) system evaluation, aimed to measure the velocity and uniformity of water distribution within the root zone.

For sensor calibration in step A, 8 WaterScout SM 100 Soil Moisture Sensors for dielectric permittivity measure^1^ were placed on 8 cellulosic sponge panels 40 × 40 × 60 mm (length × width × height). The panels were oven-dried and weighted, then saturated with water, and readings of water content and measurements of sample weight were taken after irrigation or drying operations, to determine the correlation between the readings and the effective water content for each sensor. Data were collected in an Excel file then plotted in a graphic with the best interpolation curve (coefficient of determination *R*^2^ > 0.95). Calibration curves were reported as Relative Water Content, RWC = Water content in weight (WC, g)/Sample weight (g).

In step B, one PGU unit was assembled in a plastic box 350 × 250 × 150 mm (length x width x height), containing a cellulosic sponge panel (same dimensions), equipped with two stainless steel porous tubes 12 × 8 × 330 mm (external diameter x internal diameter x length; 316LSS, 10 micron pores diameter, Applied Porous Technologies, Inc.). The porous tubes, embedded in the cellulosic sponge panel, were connected to a distribution circuit, consisting of PVC plastic tubes (10 mm diameter), in which the desired volume of deionized water, stored in a tank, was delivered through a peristaltic pump (P3680 Sigma, Millipore Corporation) and controlled through a flow meter (FHKU Flow Sensor Riels), placed along the watering line.

Four WaterScout sensors, previously calibrated, were inserted in the sponge panel, with the objective to assess the capability of the system to uniformly distribute the water, and the time needed for the water distribution within the entire volume. Subsequently, the test was conducted following a procedure similar to the one used for sensor calibration, taking sensor measurements after the injection of a predefined quantity of water, from a known initial water status until saturation.

### Phase 3: Tuber Seeds Germination and Plant Growth

Prior to starting the plant growth test, two European cultivars of potato, (*Solanum tuberosum* L.) “Avanti” (Stet Holland B.V.), and “Colomba” (HZPC Holland B.V.), were selected according to the requirements established in the ESA project MELiSSA for cultivation in BLSSs. The following features were considered: short growth cycle, compact plant size, high harvest index, wide disease resistance, nutritional quality of tubers (high dry matter and nutrient content, low levels of anti-nutritional compounds), and tuber processability (cooking type, high starch content, regular shape, thin peel).

For the germination test, certified potato tuber seeds of the two cultivars were sterilized with sodium hypochlorite water solution (NaClO 5 g l^–1^). Based on the results of the hydrologic characterization, only three substrates were used: oasis, capillary mat, and cellulosic sponge. Tuber seeds were sown in plastic boxes prepared with the selected substrates (6 tubers seeds per cultivar per substrate), then placed in germination chambers at 18°C in the darkness (Picture 1). All the substrates were covered with a layer of cotton wool as mulching, to prevent water evaporation. Substrates were moisturized constantly by spraying sterile water.

**PICTURE 1:**
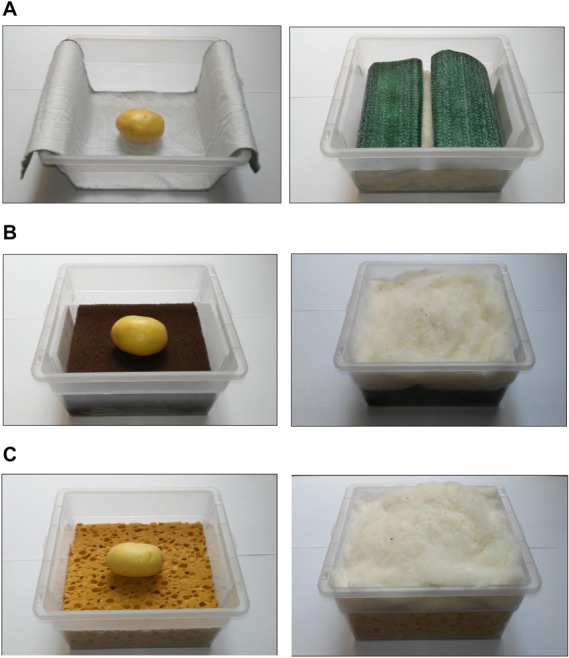
Substrate layout used for germination of potato tuber seeds in germination chamber (18°C in the darkness): **(A)** Oasis Horticubes^®^ (phenolic foam), **(B)** capillary mat (polyester fiber), and **(C)** cellulosic sponge (100% cellulose), with cotton wool as mulching to prevent water evaporation.

Based on the results of the germination test, plant growth test was carried out only with cv. “Colomba,” on cellulosic sponge, identified as the best substrate in terms of hydrologic characteristics. A layer of cotton wool was placed on top of the sponge substrate as mulching, to prevent water evaporation. Four PGUs in total were assembled as described in Phase 2, two in plastic boxes, and two made in on purpose designed Kevlar bag (both container types with 350 × 250 × 150 mm, length × width × height). All PGUs were equipped with porous tubes and with two WaterScout sensors (Picture 2) and connected to the water and nutrient distribution system. Prior to starting cultivation, all the containers and components of the distribution system were sterilized using a NaClO water solution (0.5% in vol.), while cellulosic sponge and porous tubes were sterilized in autoclave (20 min at 120°C).

**PICTURE 2:**
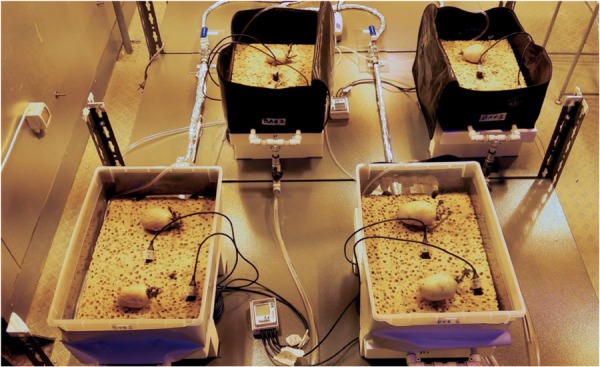
Set up of the Plant Growth Units (PGUs) of the Root Module (RM) designed in the ESA project “Precursor of Food Production Unit” (PFPU), during the Plant growth test: two ziplock bags with a zip on the top part, for tubers and roots inspection, and two plastic boxes, equipped with porous tubes, embedded in the substrate, to distribute the water or nutrient solution to the root zone.

Pre-sprouted tuber seeds were moved in the above described PGUs (2 plants per bag, 8 plants n total), and placed in a 28 m^2^ walk-in growth chamber, atmospherically open, equipped with a computer program for climatic control. The test lasted until the beginning of tuberization (60 days). The following environmental conditions were set: temperature 22/18°C (light/dark), relative humidity 60–70% (actual values actual values 21.6 ± 0.6/18.5 ± 0.3°C and 57.9 ± 8.5%, respectively; average ± standard deviation of measurements at 1 h interval), and ambient CO_2_ concentration. Light was provided by High Pressure Sodium (HPS) lamps and light intensity at the canopy level was approximately 450 μmol m^–2^ s^–1^, according to a light/dark regime of 12/12 h.

Cellulosic sponge was moistened with only deionized water until the beginning of rooting. Thereafter, fertigation with nutrient solution was alternated with irrigation, in order to prevent salt accumulation in the substrate and to keep the nutrient solution parameters in the root zone close to the target values (pH 5.5–5.8; EC 1.40–1.80 dS m^–1^). The nutrient solution was prepared according to Molders et al. (2012). Water injection took place every time the sensor readings reached the threshold value obtained in Phase 2. Samples of nutrient solutions were collected once a week for measurements of pH and EC in the root zone.

## Results

### Substrates Hydrological Characterization

Table 1 shows the physical and hydrological properties measured in the six substrates tested. Permeability tests revealed a high saturated hydraulic conductivity (K_s_) in all the substrates. In particular, the Oasis Horticubes^®^ showed the highest K_s_ value (2.82 cm/s), compared to the other substrates (range 0.27–0.88 cm/s).

**TABLE 1:** Physical and hydrological properties (Mean ± St. Error) and summary of the main characteristics as cultivation substrate for plant cultivation in Space of 3 synthetic materials, Oasis Horticubes^®^ (phenolic foam), rockwool and capillary mat (polyester fiber), and 3 organic materials, cellulosic sponge (100% cellulose), cotton and a combination of hemp and kenaf fibers.

	**Saturated hydraulic conductivity (K_s_, cm/s)**	**Bulk density (ρ, g/cm^3^)**	**Container capacity (CC, cm^3^/cm^3^)**	**Saturated hydraulic conductivity**	**Water retention**	**Structural evaluation**	**Final evaluation**
Oasis Horticubes	2.82 ± 0.09	0.021 ± 0.001	0.56 ± 0.01	High	Intermediate	Fragile and inconsistent with dispersion of dust	Good hydraulic characteristics, bad handling
Rockwool	0.70 ± 0.03	0.059 ± 0.001	0.94 ± 0.02	High	Low	Incoherent with dispersion of dust	Poor water retention, bad handling
Capillary mat	0.27 ± 0.02	0.145 ± 0.010	0.90 ± 0.03	High	Low	Compact with low thickness	Poor water retention, good handling
Cellulosic sponge	0.30 ± 0.01	0.124 ± 0.003	0.94 ± 0.02	High	Good	Elastic and resistant	Very good hydraulic characteristics, good handling
Cotton wool	0.31 ± 0.01	0.021 ± 0.001	0.33 ± 0.02	High	Intermediate	Easily adaptable and compressible	Good hydraulic characteristics, medium handling, suitable for mulching
Canapa/Kenaf	0.88 ± 0.02	0.049 ± 0.001	0.72 ± 0.03	High	Low	Extremely rigid and compact	Poor water retention, bad handling

*Handling includes the shape malleability and the safety for the crew (e.g., absence of release of dust or small particles). For Saturated hydraulic conductivity and Bulk density n = 3; for Container capacity n = 6.*

**FIGURE 3:**
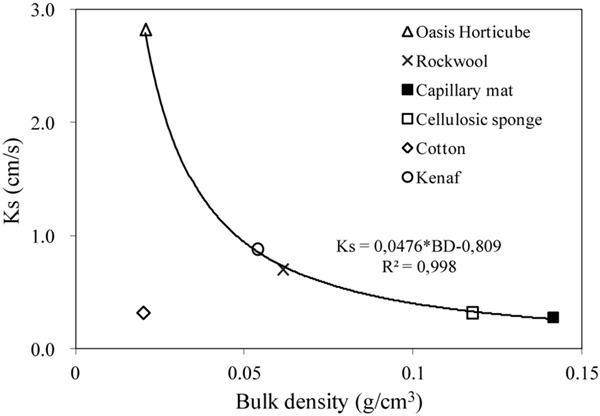
Relation between Saturated hydraulic conductivity (Ks, cm/s) and bulk density (BD, g/cm^3^) of the materials tested as substrate for plant cultivation in Space: 3 synthetic materials, Oasis Horticubes^®^ (phenolic foam), rockwool and capillary mat (polyester fiber), and 3 organic materials, cellulosic sponge (100% cellulose), cotton and a combination of hemp and kenaf fibers. Mean values of 6 replicates. Note that the cotton values (diamond gray marker) have been excluded from the computation of the parameters of the trendline.

Capillary mat and cellulosic sponge showed the highest bulk density (0.14 g cm^–3^ and 0.12 g cm^–3^, respectively). In general, for porous media an inverse relation exists between bulk density and saturated hydraulic conductivity. This was confirmed for all the examined substrates with the exception of cotton wool, which revealed a tendency toward water repellence during the permeability test (Figure 3). This behavior also contributes to explaining the low container capacity of cotton wool (0.33 cm^3^ cm^–3^) compared to the other substrates (from 0.56 cm^3^ cm^–3^ in Oasis Horticubes to 0.94 cm^3^ cm^–3^ on average in cellulosic sponge and rockwool).

**FIGURE 4:**
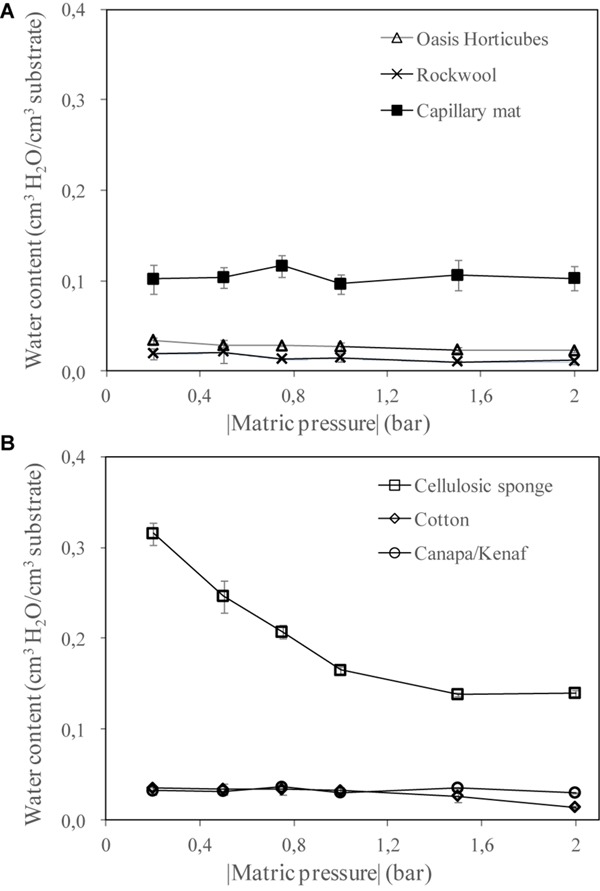
Water retention curves of the materials tested as substrate for plant cultivation in Space: **(A)** 3 synthetic materials, Oasis Horticubes^®^ (phenolic foam), rockwool and capillary mat (polyester fiber), and **(B)** 3 organic materials, cellulosic sponge (100% cellulose), cotton and a combination of hemp and kenaf fibers. Mean value ± Standard error; *n* = 6.

Figures 4A,B report the volumetric water retention curves of the inorganic and organic substrates, respectively. Results showed different hydraulic behavior in the substrates in terms of water retention at increasing suction. In particular, within the inorganic group, capillary mat reached the highest water content (about 0.1 cm^3^ cm^–3^), with no relevant changes under a different matric pressure. Similarly, the other two inorganic substrates also showed a flat pattern of water retention, with a very low water content (about 0.02 cm^3^ cm^–3^ on average). For the organic group, the cellulosic sponge revealed two interesting aspects: it showed the highest water content in the range of the matric suction tested (0.32 cm^3^ cm^–3^ at 0.2 bar), and a good gradation of water content at increasing water suction (reaching 0.14 cm^3^ cm^–3^ at 2 bar).

### Sensors Calibration and Water Distribution System Set-Up

**FIGURE 5:**
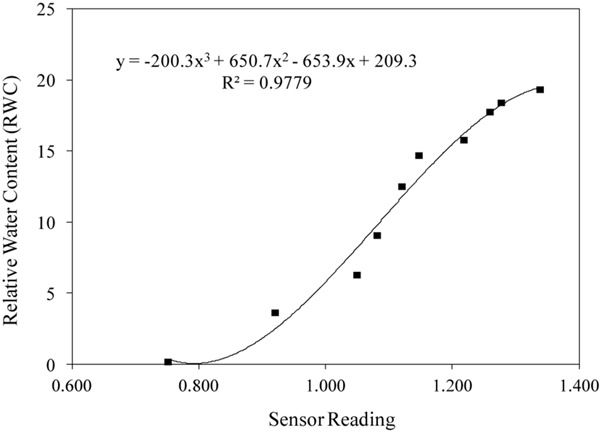
Example of calibration curves of WaterScout SM 100 Soil Moisture Sensor (www.specmeters.com/brands/waterscout/) on cellulosic sponge (Sensor nr. 8).

Table 2 reports the equations obtained in the 8 sensors during calibration, and Figure 5 shows the calibration curve obtained on cellulosic sponge for Sensor nr. 8, chosen as an example. As expected, even though all sensors showed a similar trend in the relation between the sensor reading and the substrate water content in weight, each sensor revealed its own calibration curve. Calibration curve is useful to define the lower and upper limit of readings to be used to drive irrigation during plant growth, in order to keep the substrate water content within an optimal range. Specifically, based on the interval of optimal water availability known for potato plants (−0.025 to −0.320 bar of soil moisture potential, according to Wesseling et al., 1991), the volume of water to be assured in the cellulosic sponge resulted in the range from 300 to 1100 grams per panel (Table 3). Accordingly, based on the sensor reading, the volume of water to be added in the cellulosic sponge panel at each irrigation/fertigation pulse, to restore the optimal availability, was calculated (Table 3).

**TABLE 2:** Equations of calibration curves of the 8 WaterScout SM 100 Soil Moisture Sensors (www.specmeters.com/brands/waterscout/) on a cellulosic sponge panel (250 × 350 × 150 mm, length × width × height), equipped with two 10 micron porous tubes (316LSS, 10 micron pores diameter, Applied Porous Technologies, Inc.).

**Sensor**	**Datalogger**	**Channel**	**Equation**	**Coefficient of determination**
1	1	1	y = –238.71x^3^ + 730.54x^2^–686.79x + 204.72	*R*^2^ = 0.9914
2	1	2	y = –40.51x^3^ + 133.30x^2^–103.31x + 21.91	*R*^2^ = 0.9830
3	1	3	y = –535.00x^3^ + 1484.50x^2^–1295.40x + 362.67	*R*^2^ = 0.9579
4	1	4	y = 121.99x^3^–279.15x^2^ + 229.17x–64.79	*R*^2^ = 0.9828
5	2	1	y = –23.70x^3^ + 129.52x^2^–146.81x + 47.55	*R*^2^ = 0.9899
6	2	2	y = –380.01x^3^ + 1168.90x^2^–1129.30x + 349.48	*R*^2^ = 0.9708
7	2	3	y = 241.10x^3^–703.68x^2^ + 694.59x–226.69	*R*^2^ = 0.9744
8	2	4	y = –200.30x^3^ + 650.66x^2^–653.87x + 209.29	*R*^2^ = 0.9779

**TABLE 3:** Relation between the readings of moisture sensors (WaterScout SM 100 Soil Moisture Sensor) and the water content in cellulosic sponge substrate.

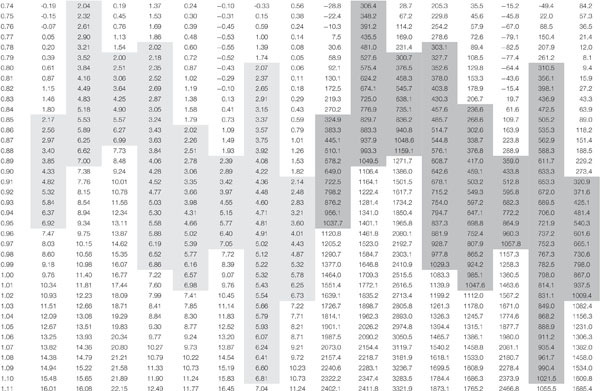

*Pale and darker gray represent the reference intervals of optimal values for potato crop in Relative water content and Total water content, respectively, to be used to identify the time and volume of the irrigation/fertigation pulse. Specifically, values below the lower limit indicate the need for water/nutrient solution injection, while values over the upper limit indicate water over-load in the substrate.*

**FIGURE 6:**
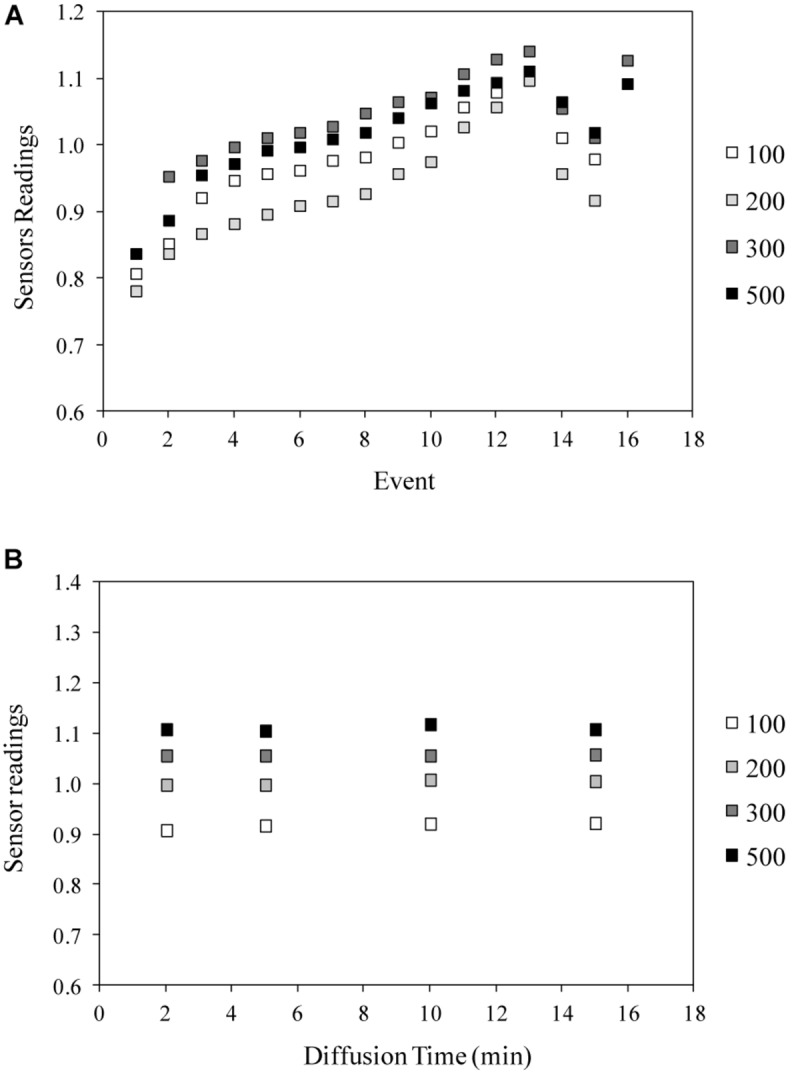
Results of the Water distribution test with WaterScout SM 100 Soil Moisture Sensor (www.specmeters.com/brands/waterscout/) in the cellulosic sponge substrate, in terms of: **(A)** sensor readings as function of water addition and depletion, and **(B)** time of water diffusion, after the addition of 100, 200, 300, or 500 ml (Sensor nr. 1). Events from 1 to 13 and event 17 represent water injections, while events 14 and 15 represent water removal.

As a successive step, the results of the water distribution test demonstrated the efficiency of the designed porous tubes system in terms of velocity and uniformity of water distribution within the sponge panel, and confirmed the good performance of WaterScout sensors in the proposed system layout (Figure 6A). Indeed, even if slight differences among the sensors were recorded, the trend for the readings and the time of reaction after water injection were similar for all the sensors, demonstrating a good water distribution within the substrate panels. In addition, this distribution was very fast; as an example, Figure 6B shows the readings after the injection of four different volumes of water (100, 200, 300, and 500 ml), after 2, 5, 10, and 15 min, in sensor nr. 1, demonstrating that each reading was stable 2 min from the injection.

Unexpectedly, saturation point measured during the distribution test was lower compared to those found during the sensor calibration (1.08 vs. 1.22, respectively).

The mulching cotton wool, weighted before starting the test and after water saturation of the cellulosic sponge, was shown to absorb water from the surface of cellulosic sponge at a negligible quantity (5% of the its weight). However, after the handling required for a periodic inspection of the system, cotton wool lost consistency and compactness, releasing fabric fibers.

### Tuber Seeds Germination and Plant Growth

All the tuber seeds of both the selected cultivars “Avanti” and “Colomba” germinated in all the substrate layouts, being kept constantly moist with deionized water (Picture 1).

Tuber seeds of “Colomba” were earlier in sprouting compared to “Avanti” and did not show relevant variability within each substrate and differences among the different substrate layouts in the time for sprouting, which varied from 12.7 to 16.2 days from sowing (Table 4). Conversely, tuber seeds of “Avanti” revealed a greater variability in the sprouting time, which ranged from 29.5 to 41.2 days, and showed a slower and incomplete sprouting on cellulosic sponge (Table 4).

**TABLE 4:** Rate of tuber-seeds sprouting (% of the total number of tubers) and Average Time of Sprouting (ATS, in days) (Mean ± St. Error) in potato cultivars “Avanti” and “Colomba” sown on 2 synthetic materials, Oasis Horticubes^®^ (phenolic foam), rockwool and capillary mat (polyester fiber), and one organic material, cellulosic sponge (100% cellulose), using cotton wool as mulching.

	**“Avanti”**		**“Colomba”**	
	**Sprouting rate (%)**	**ATS (days)**	**Sprouting rate (%)**	**ATS (days)**
Oasis Horticubes + Cotton wool	100	29.5 ± 2.3	100	16.2 ± 2.2
Capillary Mat + Cotton wool	100	32.0 ± 2.9	100	12.7 ± 0.5
Cellulosic sponge + Cotton wool	83	41.2 ± 8.3	100	15.7 ± 1.6

In the plant growth test, potato tuber seeds cv. “Colomba” pre-sprouted in a germination chamber started to root on cellulosic sponge 5 days after transplanting (DAT) on average (Picture 3). As roots deepened in the sponge substrate, a visual inspection from the bottom of transparent plastic boxes revealed a uniform development of healthy roots of a white color, with no brown area on the apexes.

**PICTURE 3:**
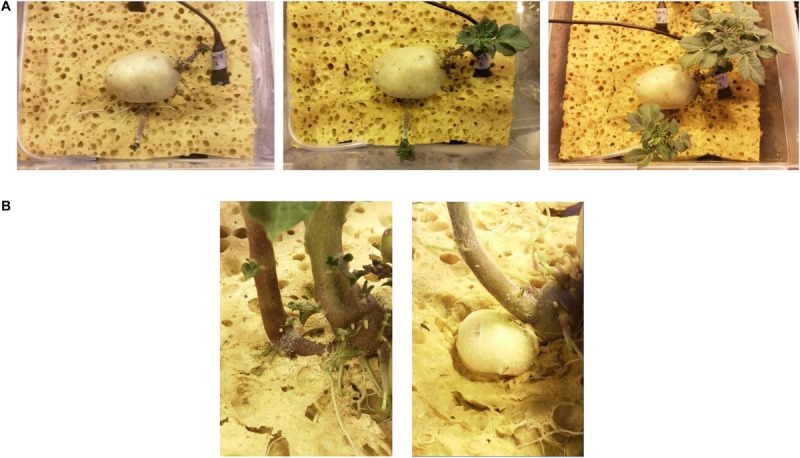
Subsequent phases of development of potato plants cv. “Colomba” grown on cellulosic sponge in growth chamber, under controlled environment: **(A)** rooting and stem and leaf development; **(B)** stolons and tubers formation.

The stolons formation began 24 DAT, in plants 32 cm high, with 8 compound leaves on average, and it was followed by the appearance of tubers, 33 DAT.

When the experiment was stopped (60 DAT), both the aerial part and the root system of potato plants were normally developed, with roots spread in the inner and bottom part of the panel and stolons and tubers at the surface layer. At this time, tubers had 3.5 cm of diameter on average.

As regards the irrigation/fertigation, the number of pulses ranged from 1 to 2 per week and the volume required to keep the water content in the above mentioned optimal interval ranged from 70 to 200 ml per plant (140–400 ml per bag), depending on the plant size and the related water uptake.

With an average of 7 samplings along the growing cycle, the mean values of pH and EC were 7.6 and 2.02 dS m^–1^, respectively. Because of the increase of pH over the target interval (5.5–5.8), in the last 3 weeks of cultivation, deionized water was acidified with nitric acid at pH 4.0 in order to better control pH fluctuations in the root zone.

Despite the preliminary disinfection of tuber seeds and cultivation devices, a fungal contamination occurred in the substrate starting from the third week of cultivation, however the pathogen did not colonize the potato plants.

## Discussion

### Substrates Hydrological Performance

The results of the physical and hydrological characterization of the proposed growing media need to be interpreted while considering their specific uses in the Root Module. As regard the capability of the tested substrates to allow the movement of water and air, the Oasis Horticubes^®^ showed a K_s_ value equivalent to a gravel soil, while those of all the other substrates were comparable to a coarse sand soil. These findings confirm that all of the substrates tested are capable of transporting the nutrient solution from the delivery system to the growing medium with a proper velocity. In addition, a good rate of gas exchange in the substrates can be expected given the high values of K_s_ and the positive correlation with k_a_, previously demonstrated (Loll et al., 1999; Iversen et al., 2001).

The relation between K_s_ and BD determined in the tested substrates represents an easy tool to predict the capacity of a growing media to transmit properly the nutrient solution and air to the root zone, only by considering bulk density, that can be determined more easily with respect to K_s_. The substrates tested manifested a proper container capacity, except the cotton wool that showed a tendency to water repellence and collapse phenomena during the permeability test. The lack of structure of cotton implies an unstable pore spaces system, while the use of porous media with prescribed and stable pore spaces is a key principle for reliable management of substrates in microgravity (Jones et al., 2005).

Another important issue is the capability of the substrate to feed the root system efficiently, even when the matric suction increases. To assess the differences between the tested substrates, the analysis of water retention curves led to the conclusion that cellulosic sponge holds the highest amount of available water, while the other substrates available revealed a poor water content even at low levels of matric suction.

In terms of air, water, and nutrient transport processes, all the selected substrates, with the exception of cotton wool, are eligible to be used as growing media in hydroponic cultivation systems on Earth. However, with the exception of cellulosic sponge, all the substrates need a frequent irrigation to maintain an adequate water status for the root extraction, due to their poor water retention (Spomer, 1994). This shortcoming doesn’t match the requirements for cultivation of tuberous plants in microgravity, which implies that substrates should be wetted with a minimum amount of energy and water.

Cellulosic sponge provides the highest water holding capacity and capillary rise thanks to the wider distribution of pore radiuses, also guaranteeing the proper air content. This is also consistent with the findings of Steinberg et al. (2005), who encouraged the use of porous media with a broader pore size distribution, given that it may reduce cluster scale effects (clusters of several particles glued by water) caused by microgravity. For these reasons, cellulosic sponge was found to be the best material as substrate for the root zone.

### Sensors Calibration and Water Distribution System Set-Up

Sensor calibration demonstrated the suitability of Waterscout SM 100 soil moisture sensors to be used in natural sponge substrate. However, care must be taken in the practical application, since each sensor has its own calibration curve, and the attempt to use average values to find one single calibration curve for all the sensors determined large errors when applied for single sensor measurements. In addition, each calibration curve is valid using the same sensor/datalogger configuration (i.e., connecting to the same datalogger and the same channel used for calibration).

A water distribution test demonstrated that the water diffusion process in the cellulosic sponge panel was fast and efficient, as a uniform humidification was reached in less than 2 min from the water injection. It is worth noting that the lower saturation point recorded in this test, compared to those measured during sensor calibration, could depend on the containment effect exerted by containers, which limited the expansion of the wetted sponge. This result will be considered in the execution of the future tests.

As expected, water and nutrient solution were not absorbed by the cotton wool mulching.

The test showed that the use of four sensors per box/bag was not required, while two sensors were sufficient to monitor the humidity level in the root zone. However, some constraints need to be considered in the porous tubes system. For instance, the test demonstrated that porous tubes work properly only when they are completely filled with water, since air determines unequal distribution of water along the porous tube, implying the need for preliminary flushing to remove air throughout the circuit.

Based on the hydraulic properties, cellulosic sponge represents a good alternative to traditional substrates for hydroponics, suitable for distributing and holding water and nutrient solution. Conversely, cotton wool used as mulching revealed a poor structure stability, implying risks for the crew safety (release of particles in the air).

### Tuber Seeds Germination and Plant Growth

Time for tuber seeds germination was different between the two potato cultivars “Avanti” and “Colomba.” This result can be determined by genotypic reasons as well as by agronomical aspects, including the conditions of mother-plants cultivation and the preparation protocol for sale and transport of tuber seeds adopted by the different breeders providing the tubers seeds of the two cultivars (Frusciante and Roversi, 2011).

In “Colomba” plants cultivated on cellulosic sponge in a growth chamber, under HPS lamps, the time for the beginning of tuberization was similar to those observed in the same cultivar grown on a peat-based substrate in phytotron, under white fluorescent light and similar conditions of temperature and relative humidity (Paradiso et al., 2018).

The plant growth of potato on cellulosic sponge in a controlled environment followed a normal pattern for the crop; plants developed well and did not show any symptoms of unbalanced mineral nutrition. This latter result demonstrates that the strategy of water and mineral nutrition adopted in the experiment, alternating irrigation and fertigation pulses to contain the EC and pH fluctuations in the root zone, was efficient in guaranteeing non-limiting levels of water and nutrients in the growing medium for plant growth.

Cultivation protocol used in the plant growth test with “Colomba” on cellulosic sponge allowed the obtainment of healthy potato plants and the completion of the tuber-to-tuber cycle. In addition, the distribution of roots and of stolons and tubers, in the inner and bottom part of the panel and at the surface layer, respectively, allows for an efficient inspection during the experiment and an easy staggered harvest of tubers.

Despite the sterilization treatment before the test, cellulosic sponge experienced pathogen infection, requiring medical treatments. Fungal infection represents a real threat in the cultivation layout proposed, because of the constant humidity and availability of nutrients for pathogens due to the considerable holding capacity of cellulosic sponge. However, it is worth noting that, even in the presence of fungi, roots and tubers were not colonized and plants remained healthy, indicating the high specificity needed by the pathogens for the cellulose-based substrate. Nevertheless, sterilization, microbial analysis, and definition of chemical treatments during plant growth are sensitive items, also considering that a deep tuber disinfection is not possible (Frusciante and Roversi, 2011).

## Conclusion

Based on the analysis of the hydrologic performance, in terms of air and water transport and water retention capacity, cellulosic sponge is the best candidate substrate for plant cultivation in the PFPU Root Module among the tested materials.

WaterScout water sensor, designed for measurements of water content in natural soil and peat-based substrates, is suitable for monitoring the water status in cellulosic sponge and to drive irrigation and fertigation management in the Root Module.

The designed distribution system, based on a porous tubes circuit, is able to provide water or nutrient solutions in a timely and uniform way in cellulosic sponge. Based on the definition of lower and upper limits of the sensor readings, it is possible to define an efficient irrigation strategy, while also preventing drying or over-loading conditions in the root zone.

Cellulosic sponge is suitable to support potato plants throughout a tube-to-tuber cycle, however, it revealed to be sensitive to fungal infection. In the view of future experiments in the same breadboard, pre-treatments with a wide-range of antifungal products will be considered before sowing.

During plant cultivation, the fertigation strategy adopted, based on the alternation of supply with deionized water or nutrient solution, was efficient in preventing salt accumulation in cellulosic sponge and alkalinization of the rhyzosphere. Fertigation management in future experiments could be improved by using the next generation sensors, allowing also the measurement of pH and the electrical conductivity.

The overall analysis of our results indicates that the proposed design of the Root Module, including a Kevlar bag as a container, cellulosic sponge as cultivation substrate, and a porous tube based system for water and nutrient solution distribution, was efficient in guaranteeing the proper conditions in the root zone to obtain healthy plants, able to complete the tuber-to-tuber cycle. However, it is worth noting that results from ground-based experiments do not allow us to foresee either the substrate or the plant behavior in altered gravity. Consequently, as mentioned, all our findings need to be confirmed in flight experiments.

## Data Availability Statement

All datasets generated for this study are included in the article/supplementary material.

## Author Contributions

SD and RF conceived the project. RP performed the plant growth test and assembled the first draft of the manuscript. AP and MP performed the hydrological characterization of the substrates. AC and SS realized the water distribution system and carried out the sensors calibration. MG contributed to the plant growth test. All the authors contributed to writing the manuscript.

## Conflict of Interest

AC, SS, and RF was employed by company Telespazio S.p.A., Italy.

The remaining authors declare that the research was conducted in the absence of any commercial or financial relationships that could be construed as a potential conflict of interest.

## Footnotes

1 www.specmeters.com/brands/waterscout/
